# Head-to-Head Comparison of Etanercept vs. Adalimumab in the Treatment of Ankylosing Spondylitis: An Open-Label Randomized Controlled Crossover Clinical Trial

**DOI:** 10.3389/fmed.2020.566160

**Published:** 2020-10-30

**Authors:** James Cheng-Chung Wei, Hsi-Kai Tsou, Pui-Ying Leong, Chia-Yin Chen, Jin-Xian Huang

**Affiliations:** ^1^Department of Rheumatology & Immunology, Chung Shan Medical University Hospital, Taichung, Taiwan; ^2^Institute of Medicine, Chung Shan Medical University, Taichung, Taiwan; ^3^Graduate Institute of Integrated Medicine, China Medical University, Taichung, Taiwan; ^4^Functional Neurosurgery Division, Neurological Institute, Taichung Veterans General Hospital, Taichung, Taiwan; ^5^Department of Rehabilitation, Jen-Teh Junior College of Medicine, Nursing and Management, Miaoli County, Taiwan; ^6^Division of Rheumatology, Department of Medicine, The University of Hong Kong-Shenzhen Hospital, Shenzhen, China

**Keywords:** ankylosing spondylitis, etanercept, adalimumab, efficacy, safety

## Abstract

**Background:** Anti-tumor necrosis factor biological agents had been proved to have a dramatic effect in ankylosing spondylitis (AS). We aimed to determine the efficacy and safety of crossover effects of adalimumab vs. etanercept in AS patients.

**Methods:** A randomized, open-label crossover study was done in patients with active AS. Patients were randomized into two sequence groups, etanercept first (treatment arm) vs. adalimumab first (control arm) 8 weeks and then switched over for another 8 weeks. The primary endpoints were the difference of the Bath AS activity index and AS disease activity score (ASDAS)crp at week 16. Secondary endpoints were ASDASesr, ASAS20, and ASAS40 response rates and the proportion of patients achieving ASDAS inactive disease and low disease activity at weeks 8 and 16. Patient global assessment and preference was grading on a numerical scale.

**Results:** A total of 21 patients were screened, and 19 of them were randomly allocated into the treatment arm (*n* = 9) and control arm (*n* = 9). At baseline, age, sex, Bath AS activity index, and ASDAS of both arms were comparable (*p* > 0.05). Both arms showed dramatic improvement, whereas no significance was observed between the changes of ASDAScrp (0.90 ± 1.39 vs. 1.24 ± 1.40 at week 8, *p* = 0.612; 1.02 ± 1.22 vs. 1.26 ± 1.44 at week 16, *p* = 0.707, respectively). ASAS20 and ASAS40 response rates were also comparable at week 8 (33 vs. 44%, *p* = 1.000; 22 vs. 22%, *p* = 1.000) and week 16 (22 vs. 22%, *p* = 1.000; 22 vs. 22%, p = 1.000), respectively. Both arms were well-tolerated without a serious adverse event. Adalimumab was relatively more favorable by patients in both arms, with a total mean grading score of 0.4 (−5–5, *p* = 0.218).

**Conclusion:** Etanercept and adalimumab can both dramatically improve disease activity in 16 weeks. Crossover administration of etanercept and adalimumab revealed comparable efficacy and safety.

**Trial Registration:** The protocol was approved by the Institutional Review Board with the register CS08019 from Chung Shan Medical University Hospital (CSMUH), Taichung, Taiwan and registered at ClinicalTrials.gov Protocol Registration and Results System: NCT02489760.

## Introduction

Ankylosing spondylitis (AS) is chronic inflammatory arthritis causing back pain, peripheral arthritis, and enthesitis. Anti-tumor necrosis factor (TNF) biological agents, including etanercept (ETN) (1, 2), infliximab (IFX) (3, 4), adalimumab (ADA) (5, 6), certolizumab (7, 8), and golimumab (9, 10) had been proved to have dramatic anti-inflammatory and immunomodulatory effects in AS. Emerging evidence of other biotechnological drugs, including interleukin 17 inhibitors (11, 12) and Janus kinase inhibitors (13), has also been raised in AS recently. Nonetheless, ETN and ADA are considered cost-effective in the treatment of AS (14, 15).

Switching would be useful in AS patients due to the different chemical structures and mechanisms of action of TNFi. No prospective randomized controlled switch study between ETN and ADA has been done so far. In the literature, there are till now published systemic reviews and mono- or multicenter retrospective studies of real-life that have indicated that switching would be beneficial for the majority of patients with AS who failed the initial TNFi treatment, but there are no available data of open-label randomized controlled trial (RCT) focused on the comparison between these two originator TNFi (16, 17). Few trials have so far published comparative data between original and biosimilar TNFi in AS and other spondyloarthritis (18–21). Switching to ETN after IFX treatment escape restores the clinical response in most patients (22). Thus, we conducted this open-label randomized controlled crossover study in ETN- and ADA-treated AS patients to analyze the effect of switching TNFi.

## Materials and Methods

### Trial Design

The study was a phase IV, crossover, open-label, randomized controlled clinical trial, carried out in Chung Shan Medical University Hospital (CSMUH), Taichung, Taiwan. The study was reviewed and approved by the CSMUH Institutional Review Board (CS08019). A written informed consent form was obtained before subjects were enrolled. The study was registered in ClinicalTrials.gov with identifier number NCT02489760.

### Patients

Adults patients, aged between 18 and 70 years, classified according to the 1984 Modified New York Criteria for AS, were enrolled (23). At randomization, patients should be on stable background therapy as non-steroid anti-inflammatory drugs for at least 2 weeks, glucocorticoid for 4 weeks, and disease-modifying antirheumatic drugs, including sulfasalazine (SSZ) and methotrexate (MTX), for 8 weeks. All patients were biologics-naive. Exclusion criteria were participants with serum creatinine ≥3.0 mg/dl and/or alanine aminotransferase (serum glutamic pyruvic transaminase) ≥5 times the laboratory's upper limit of normal. Pregnant or breast-feeding women, participants with active tuberculosis infection, and those who do not meet the indication of using ETN and ADA were also excluded from the study.

### Interventions

Eligible patients were randomized equally into two arms. The treatment arm received ETN (Enbrel) 25 mg subcutaneously twice a week for 8 weeks then switched to ADA (Humira) 40 mg subcutaneously biweekly for another 8 weeks. The control arm received ADA 40 mg subcutaneously biweekly for 8 weeks. At week 8, the control arm switched to ETN 25 mg subcutaneously biweekly for another 8 weeks. Evaluations will be performed at baseline and weeks 4, 8, 12, and 16. All concomitant treatments were maintained at a stable dose during the whole 16 weeks' study duration.

### Endpoints

The primary endpoints of this study were the difference of the Bath AS activity index (BASDAI) and AS disease activity score (24) (ASDAS)crp at week 16. Secondary endpoints included ASDASesr, ASAS20, and ASAS40 (25, 26), the proportion of patients achieving ASDAS inactive disease (ID), and low disease activity (LDA) (27) at weeks 8 and 16. Laboratory assessment of inflammation with hypersensitive C-reactive protein, immunoglobulin A, and erythrocyte sedimentation rate as well as serum creatinine, alanine aminotransferase (serum glutamic pyruvic transaminase), and complete blood count were also recorded. In addition, the patient preference scale was assessed in our evaluation. Patients graded their preference of ETN and ADA on a scale of −5–5 points. If the patient favored ETN (Enbrel), his/her graded score was from −5 to 0. If the patient favored ADA (Humira), his/her graded score was from 0 to 5. A lower score represented a preference for Enbrel, and a higher score represented a preference for Humira.

### Safety Evaluation

All patients who received at least 1 dose of test medicine were evaluated for safety. The following variables were assessed: physical examination/vital signs, hematology and chemistry profiles, urinalysis, assessment of extra-spinal and extra-articular involvement, premature withdrawal, adverse events, and serious adverse events during the study.

### Statistical Analysis

Descriptive statistics was used to summarize the continuous variables. Frequency and proportion were used to summarize the categorical variables. The primary analysis was performed based on the intent-to-treat population. Frequency of dropouts, premature termination of study medication, and withdrawal were provided and summarized. The last-observation-carried-forward approach was used to evaluate missing data. All statistical tests were two-sided, which was evaluated at 5% level significance.

### Sample Size Estimation

This is a single-center open-labeled pilot study. Let us assume that the BASDAI scores of the treatment group (μ1) were reduced to 2 from 4 and the BASDAI scores of the control group (μ0) were reduced to 3.5 from 4. A two-sided test was used, with type I error (α) = 0.05, statistical power (1-β) = 0.8, and standard deviation = 2. The expected dropout rate was 10% in this research. Finally, the study required only 14 subjects in each group to achieve 95% power. The targeted sample size for the study was 30 patients. However, the study was terminated at 19 because of difficulty in patient enrollment.

## Results

### Participants

**Figure 1:**
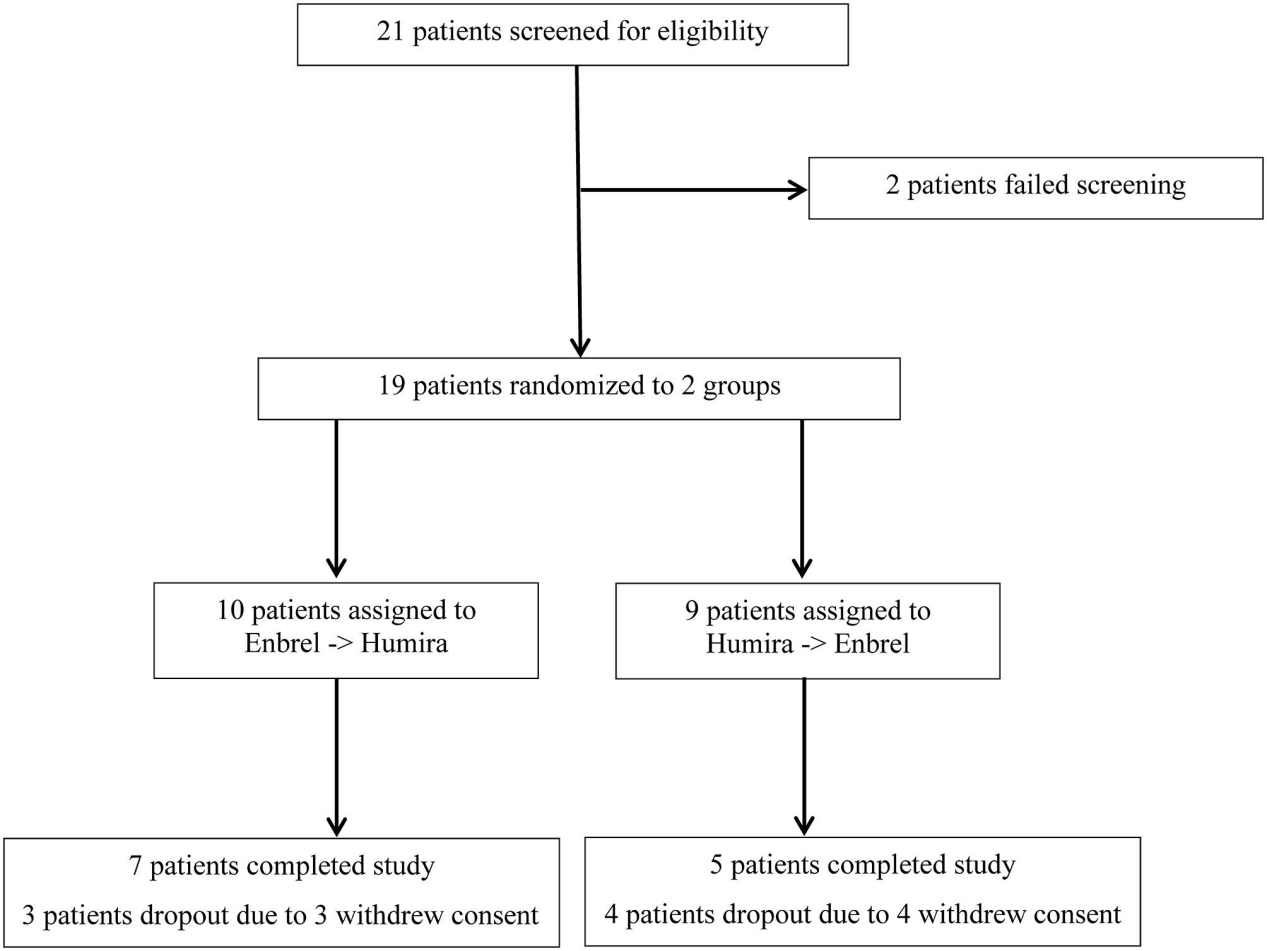
Screening procedure for an eligible patient enrolled for the treatment arm and the control arm.

A total of 21 participants were screened, and 19 were eligible and randomized into two groups. At the very beginning, 10 patients were assigned to the Enbrel→Humira group (1 patient withdrew consent before injection), and 9 patients were assigned to the Humira→Enbrel group. Therefore, nine patients for each group were included in the final analysis (Figure 1). In the Enbrel→Humira group, three patients dropped out, including two dropouts at 30 days and 53 days due to cost that was unable to be covered by medical insurance and one dropout at 93 days for unknown reasons. In the Enbrel→Humira group, four patients dropped out, including one dropout at 77 days due to inefficacy, one dropout at 63 days with treatment change to Enbrel, one dropout at 100 days due to cost that was unable to be covered by medical insurance, and one dropout due to screen failure. Baseline values for various disease activity and other measures were similar at the start of the first drug administration (Table 1). At baseline, age- and sex-matched for two arms were enrolled. HLA-B27 positivity values were 88.89% in the Enbrel→Humira group and 71.43% in the Humira→Enbrel group and 81.25% in total. For concomitant treatment, three patients (one in the Humira→Enbrel group and two in the Enbrel→Humira group) were on MTX with an average dosage of 11.67 mg per week. Thirteen patients (four in the Humira→Enbrel group and nine in the Enbrel→Humira group) were on SSZ with an average dosage of 1.62 g per day. Only two patients in the Enbrel→Humira group were in combination treatment of MTX and SSZ. Totally one patient discontinued MTX, and two patients discontinued SSZ in Humira→Enbrel. BASDAIs were 4.67 ± 2.71 for the treatment arm and 4.25 ± 2.84 for the control arm (*p* > 0.05). ASDAScrps were 2.02 ± 1.34 for the treatment arm and 2.05 ± 1.38 for the control arm (*p* > 0.05).

**Table 1:** Baseline demographics characteristics.

	**Enbrel→Humira**	**Humira→Enbrel**	***p*-value**
	**(*n* = 9)**	**(*n* = 9)**	
Age (years)^a^	39.44 ± 11.20	36.00 ± 7.57	0.456
Sex (male %)	5 (55.56%)	7 (77.78%)	0.720
NSAIDs (%)	8 (88.9%)	6 (66.7%)	0.576
DMARDs(%)	8 (88.9%)	6 (66.7%)	0.294
Weight (kg)^b^	59.00 (17.00)	61.00 (16.00)	0.544
Height (cm)^a^	164.00 ± 11.54	165.00 ±6.95	0.827
SBP (mmHg)^a^	130.40 ± 15.56	116.30 ± 14.18	0.062
DBP (mmHg)^a^	76.22 ± 11.33	75.00 ± 8.40	0.798
MBP (mmHg)^a^	94.29 ± 12.05	88.78 ± 8.12	0.272
WBC (10^3^/μl)^a^	7.19 ± 2.71	7.06 ± 0.75	0.896
RBC (10^4^/μl)^a^	460.00 ± 52.88	481.70 ± 57.20	0.416
Hb (gm/dl)^a^	13.29 ± 1.50	12.71 ± 1.65	0.449
Ht (%)^a^	40.67 ± 3.88	39.84 ± 3.70	0.652
MCV (fl)^b^	88.20 (6.70)	86.20 (5.30)	0.345
MCH (Pg)^b^	28.70 (3.90)	28.20 (2.40)	0.345
MCHC (g/dl)^b^	32.60 (0.60)	32.50 (1.10)	0.462
Platelet (10^3^/μl)^a^	264.56 ± 54.19	250.00 ± 73.03	0.638
GPT (IU/l)^b^	14.00 (6.00)	13.00 (10.00)	1.000
Creatinine (mg/dl)^a^	0.83 ± 0.10	0.84 ± 0.17	0.870
HS-CRP (mg/dl3)^b^	0.84 (2.95)	0.26 (0.53)	0.728
ESR (mm/h)^b^	16 (28)	18 (20)	0.862
IgA^b^	222.00 (55.00)	255.00 (224.00)	0.862
PGA^a^	6.06 ± 1.98	4.44 ± 1.24	0.055
PtGA^a^	5.67 ± 2.24	4.67 ± 2.65	0.399
BASDAI^a^	4.67 ± 2.71	4.25 ± 2.84	0.752
BASFI^b^	2.40 (3.35)	5.30 (1.50)	0.303
BAS-G^a^	4.95 ± 3.03	5.17 ± 2.59	0.873
ASDAScrp	2.02 ± 1.34	2.05 ± 1.38	0.971
ASDASesr	1.53 ± 0.99	1.61 ± 1.04	0.865

a *mean ± SD*.

b *median (IQR)*.

*NSAIDs, non-steroidal anti-inflammatory drugs; DMARDs, disease-modifying antirheumatic drugs; SBP, systolic blood pressure; DBP, diastolic blood pressure; MBP, mean blood pressure; WBC, white blood count; RBC, red blood count; Hb, hemoglobin; Ht, hematocrit; MCV, mean corpuscular volume; MCH, mean corpuscular hemoglobin; MCHC, mean corpuscular hemoglobin; GPT, alanine aminotransferase; HS-CRP, hypersensitive C-reactive protein; ESR, erythrocyte sedimentation rate; PGA, Physician Global Assessment; PtGA:Patient Global Assessment; BASDAI, Bath Ankylosing Spondylitis Activity Index; BASFI, Bath Ankylosing Spondylitis Functional Index; BAS-G, Bath Ankylosing Spondylitis Patient Global Score; ASDAS, Ankylosing Spondylitis Disease Activity Score*.

### Efficacy Endpoints

**Figure 2:**
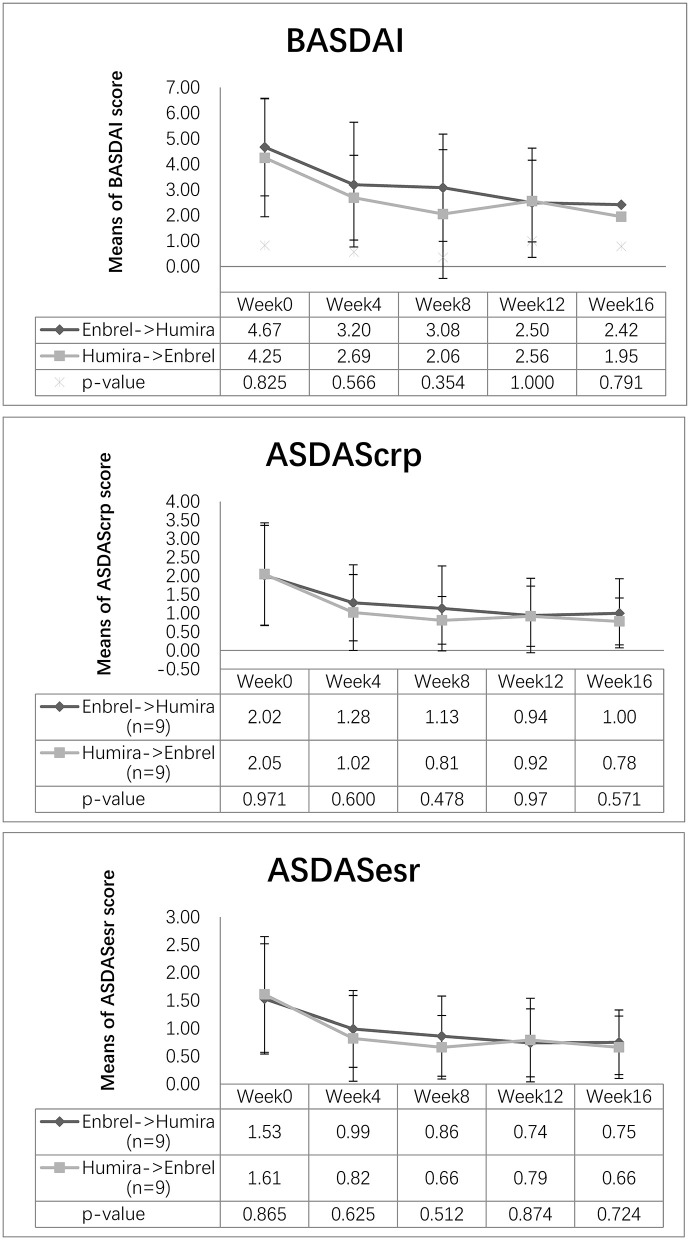
Changes in disease activity scores for the two arms. Descending trend was noticed in BASDAI, ASDAScrp, and ASDASesr in both the treatment arm and the control arm. Outcome measurements were comparable for the three disease activity scores at weeks 8 and 16 (*p* > 0.05).

The mean values of ASDAScrp dropped from 2.02 to 1.13 at week 8 and remained 1.00 at week 16 for the treatment arm. Similarly, this value dropped from 2.05 to 0.81 at week 8 and continued to decrease to 0.78 at week 16 for the control arm. The difference of ASDAScrp between two arms was not significant at weeks 8 and 16, with *p*-values of 0.478 and 0.571, respectively (Figure 2). ASDASesr also showed a similar trend of decreasing from baseline to the last visit for both arms without a significant difference between the two arms (see more details in Figure 2).

The mean value of BASDAI dropped at week 8 for both arms, decreasing from 4.67 to 3.08 for the treatment arm and from 4.25 to 2.06 points for the control arm, without significant difference between the two arms (*p* = 0.354). At week 16, it continued to decrease to 2.42 points for the treatment arm and 1.95 points for the control arm, whereas the difference between the two arms was not significant (*p* = 0.791) (Figure 2).

**Figure 3:**
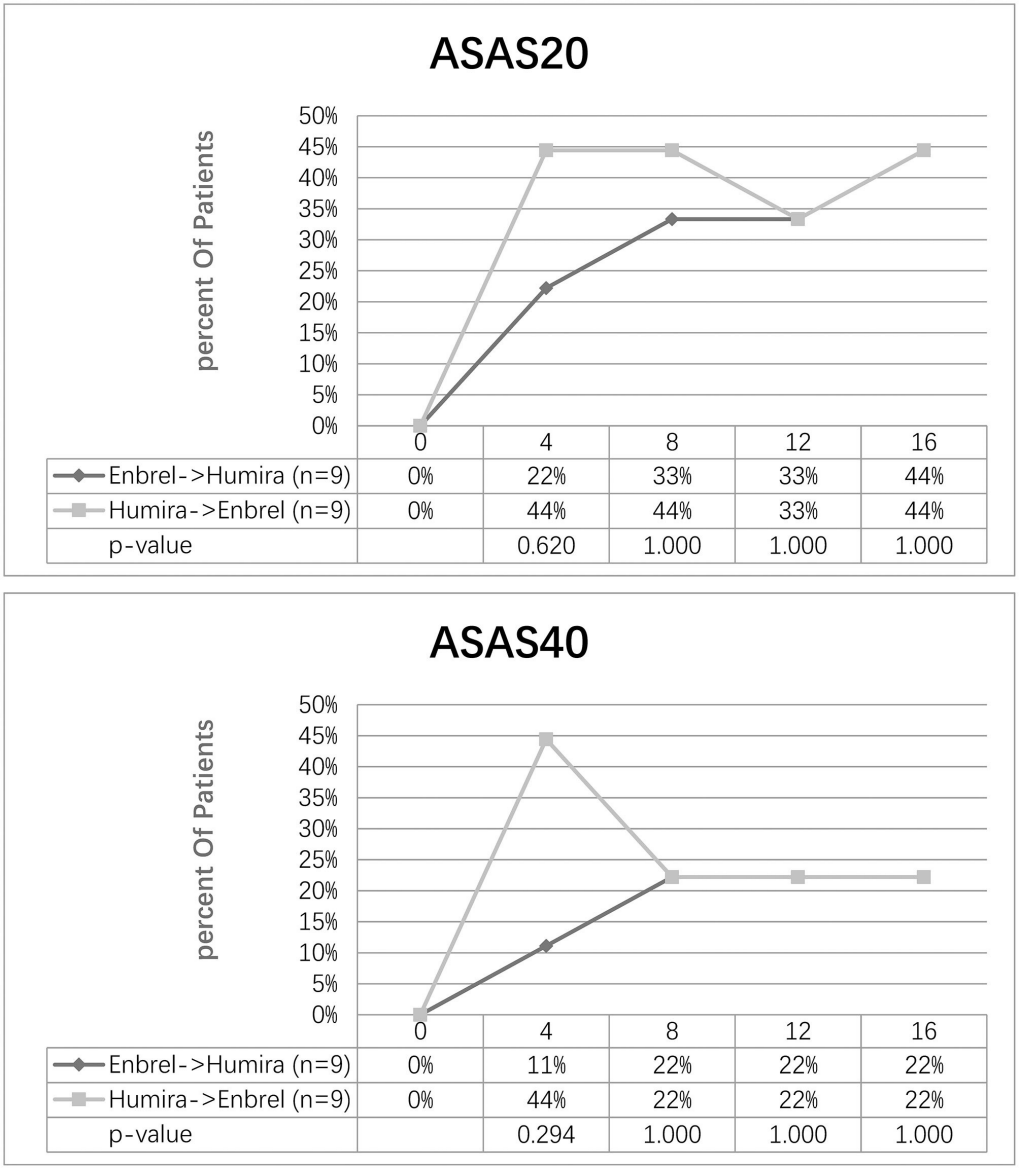
Clinical response evaluated by ASAS20 and ASAS40 for the two arms. Percentage of patients achieving ASAS20 and ASAS40 at five-time visits from baseline to week 16 was shown.

At week 8, the ASAS20 response was achieved in 33% of the participants in the treatment arm and 44% of the participants in the control arm. Nonetheless, 44% of the participants for the two arms achieved ASAS20 at week 16. At week 16, the ASAS40 response was achieved in 44% of the participants in both arms. Furthermore, 22% of the participants in both arms achieved ASAS40 at week 16 (Figure 3).

**Figure 4:**
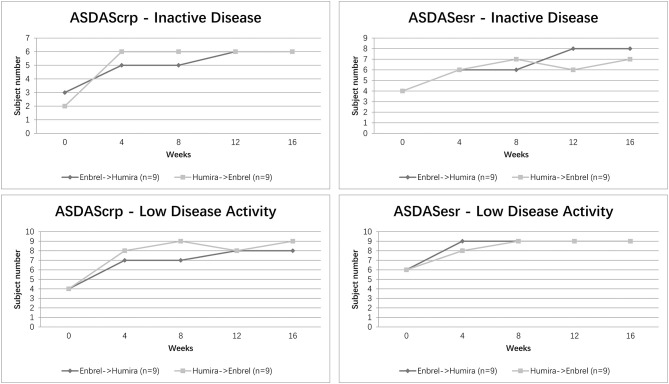
Clinical efficacy assessed by ASDAS ID/LDA status for the two arms. Percentage of patients achieving ASDAScrp and ASDASesr defined ID/LDA at five-time visits from baseline to week 16 was shown.

The proportion of patients achieving ASDAS ID/LDA at different time visits is shown in Figure 4. Overall, 5 (56%) patients in the treatment arm and 6 (67%) patients in the control arm showed an ASDAScrp ID status at week 8. Nevertheless, 6 (67%) for both arms reached ID at week 16. For ASDASesr, 6 (67%) patients in the treatment arm and 7 (78%) patients in the control arm achieved ID at week 8, whereas 8 (89%) patients in the treatment arm and 7 (78%) patients in the arm remained in ID at week 16 on the contrary. LDA for ASDAScrp was achieved in 7 (78%) patients in the treatment arm and 9 (100%) patients in the control arm at week 8 and an ascending rate of 8 (89%) patients in the treatment arm and remained 9 (100%) patients in the control arm at week 16. Even better for ASDASesr, 9 (100%) patients in both arms reached LDA at week 8 and remained at week 16 (Figure 4).

### Patient Preference

**Figure 5:**
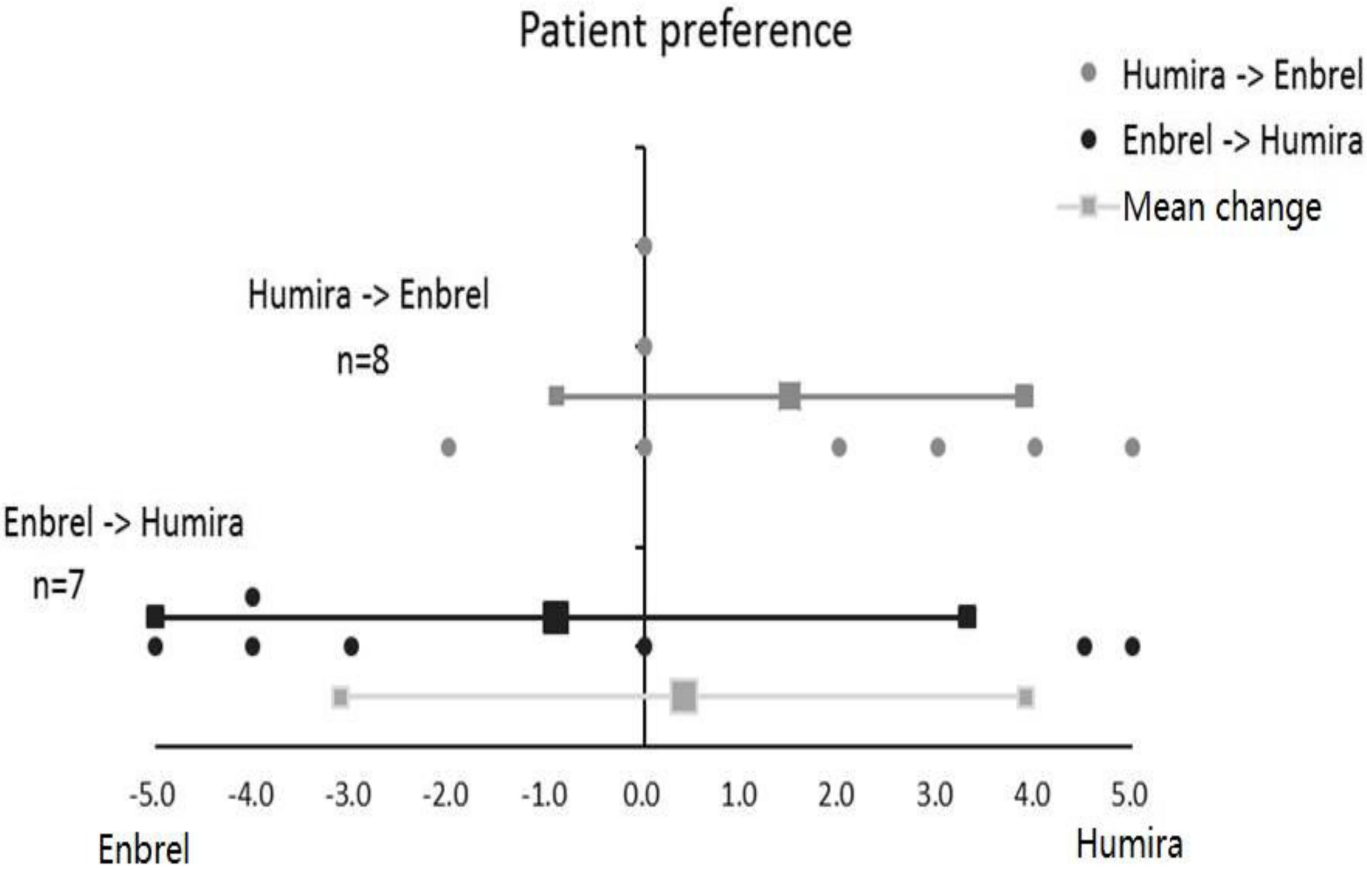
Patient preference for Enbrel and Humira in the treatment arm and the control arm. Totally seven patients were graded in the treatment arm, and eight patients were graded in the control arm.

According to the degree of patient preference, participants in the treatment arm favored more for ETN with a grading score of 0.9, and participants in the control arm favored more for ADA with a grading score of 1.5. On average, all participants in the two arms favored more for ADA with a total mean grading score of 0.4, whereas the difference between the treatment arm and control arm for patient preference grading was not significant (*p* = 0.218) (Figure 5).

### Laboratory Measurements

There were no clinically significant changes in laboratory parameters noted over the 16 weeks. The difference of inflammatory biomarkers, including hypersensitive C-reactive protein, immunoglobulin A, and erythrocyte sedimentation rate for the two arms, was not significant for the five-time visits (*p* > 0.05) (see Supplementary Materials).

### Safety Evaluation

In total, 37 adverse events were reported, including eight influenzas, five headaches, three skin itching, fatigue, and congesting eyes, two impaired liver function, oral ulcer and alopecia, one xerostomia, rhinitis, diarrhea, soreness, and swelling of bilateral hip joints, laceration of the right knee joint, insomnia, and hypertension. No severe adverse event was reported.

## Discussion

To the best of our knowledge, this study is the first to provide evidence for the clinical efficacy of crossover effects in ETN and ADA in AS. Patients in two arms both have a dramatic reduction in disease activity, as assessed by comparable change in BASDAI and ASDAS from baseline to week 16 and the non-significant difference for ASAS responses and proportion of patients achieving ASDAS ID/LDA.

TNFi seemed to be equipotent in the treatment of AS. No particular TNFi was recommended as the preferred choice for AS with a moderate level of evidence (28). TNFi switching between ETN and ADA and vice versa have been reported in several studies (29–31). A recent systemic review indicated that switching would be beneficial for most patients with AS who failed the initial TNFi treatment (32). No concrete evidence exists to support the equivalent efficacy of ETN and ADA in the treatment of AS. Thus, our crossover study further proved the equipotent of these two drugs in the treatment of AS.

The treat to target concept (33) in AS was introduced, and the main treatment target was defined as remission, with the alternative target of LDA. The proportion of ASDAS ID/LDA was similar for the two arms, indicating that the treatment target was achieved in such a short period in this specific disease setting.

The interesting point we would like to highlight in our study is the analysis of patient preference using a grading scale. Perhaps, the choice to use the patient preference scale as a variable of effectiveness is debatable because it is a non-objective measurement index and usually used to evaluate another type of objective: the compliance. It has been proved that efficacy, safety, cost, and convenience would all count in patient preference (34). It was recommended that patient preference for the dosing frequency and administration route of a specific TNFi should be weighed, and treatment should be a shared decision with the patient (33). The patient seemed to favor biologics with less frequent administration, as ADA was more convenient subcutaneously with biweekly injection compared with ETN with a once-weekly injection. Patient preference might, to some extent, reflect on drug switching, drug survival, and retention. Switching seemed to be more prominent in IFX-treated patients. Also, ETN and ADA were superior to IFX in drug survival. Intravenous administration of IFX might probably contribute to the lower drug survival. Retention was greatest with ADA, followed by ETN and IFX (35). Inflammatory phenotype, comorbidities, and socioeconomy affected drug retention (36). Patient preference data can be used to aid clinical decisions of TNFi choice.

Another measure of effectiveness is the reduction of bone marrow edema (BME) of the sacroiliac joints (SIJs) at MRI (37). Because this is a head-to-head crossover open-label RCT study, the improvement of BME at SIJ MRI would be an interesting secondary endpoint. In a recent study with AS, a quick decrease of BME in SIJ predicts better treatment response to ETN in 6 months (38). On the contrary, MRI is unable to predict clinical response to TNF-α inhibitor in AS patients within 14 weeks (39). Currently, using MRI of the SIJs as an outcome parameter in AS clinical trials evaluating the efficacy of treatment is not a validated approach (40). The observation time of our study was merely 16 weeks in total, relatively short as compared with previous studies.

Although the level of antidrug antibody was not detected in our study, it has been reported that the presence of neutralizing antibodies is associated with lower serum levels of the anti-TNF-α biologics, leading to lower efficacy and higher withdrawal rate (41). A high antidrug antibody level can avoid compromising efficacy, especially with the concomitant treatment with MTX (42). The underlying mechanism was identified as the pharmacodynamic effect of MTX via the lowering of immunogenicity and immunogenicity-mediated clearance of antidrug antibodies (43). Monitoring of the antidrug antibody level at different time visits could be served as a tool for efficacy evaluation and dosage adjustment in future studies.

Factors such as female sex, use of steroids, persistently high inflammatory levels, Bath Ankylosing Spondylitis Functional Index, and BASDAI indices were found to be negative predictors of treatment response (44, 45). Also, both ETN and ADA showed better retention of treatment when compared with IFX (46). It would be of great value to analyze the predictors of treatment response and drug survival in a head-to-head comparison between these two drugs with a larger sample size.

There were some limitations to our study. This study was underpowered for a direct comparison of ETN with ADA because of the limited sample size. Moreover, this RCT is a single-center open-labeled study design without double-blinded to patients, and physicians might introduce information bias. Additionally, although a crossover study between ETN and ADA showed dramatic effectiveness and well-tolerability in this study, short-term evaluation without follow-up might not reflect the long-term effect. Meanwhile, MRI was not selected as an endpoint due to short observation time. Larger and longer-term studies with objective outcome measures are needed to confirm our findings.

## Conclusion

ETN and ADA can both dramatically improve disease activity in 16 weeks. Crossover administration of ETN and ADA revealed comparable efficacy and safety.

## Data Availability Statement

All datasets presented in this study are included in the article/Supplementary Material.

## Ethics Statement

The studies involving human participants were reviewed and approved by Chung Shan Medical University Hospital (CSMUH) institutional review board. The patients/participants provided their written informed consent to participate in this study.

## Author Contributions

JW was the coordinating investigator. JW, P-YL, and C-YC contributed to study conduct and/or data collection. P-YL and C-YC analyzed and/or interpreted the data. JW, H-KT, and J-XH collaborated in the drafting and critical revision of the manuscript. All authors approved the final version of the manuscript and vouch for the accuracy of the analyses and the fidelity of the study to the protocol.

## Conflict of Interest

The authors declare that the research was conducted in the absence of any commercial or financial relationships that could be construed as a potential conflict of interest. The reviewer M-CW declared a shared affiliation, though no other collaboration, with one of the author, H-KT, to the handling editor.

## References

[1] Brandt J Khariouzov A Listing J Haibel H Sörensen H Grassnickel L. Six-month results of a double-blind, placebo-controlled trial of etanercept treatment in patients with active ankylosing spondylitis. Arthritis Rheum. (2003) 48:1667–75. 10.1002/art.11017 12794835

[2] Davis JC van der Heijde D Braun J Dougados M Cush J Oclegg D. Recombinant human tumor necrosis factor receptor (etanercept) for treating ankylosing spondylitis: a randomized, controlled trial. Arthritis Rheum. (2003) 48:3230–6. 10.1002/art.11325 14613288

[3] Brandt J Haibel H Cornely D Golder W Gonzalez J Reddig J. Successful treatment of active ankylosing spondylitis with the anti-tumor necrosis factor alpha monoclonal antibody infliximab. Arthritis Rheum. (2000) 43:1346–52. 10.1002/1529-0131(200006)43:6<;1346::AID-ANR18>;3.0.CO;2-E 10857793

[4] Braun J Brandt J Listing J Zink A Alten R Golder W. Treatment of active ankylosing spondylitis with infliximab: a randomised controlled multicentre trial. Lancet. (2002) 359:1187–93. 10.1016/S0140-6736(02)08215-6 11955536

[5] Haibel H Rudwaleit M Brandt HC Grozdanovic Z Listing J Kupper H. Adalimumab reduces spinal symptoms in active ankylosing spondylitis: clinical and magnetic resonance imaging results of a fifty-two-week open-label trial. Arthritis Rheum. (2006) 54:678–81. 10.1002/art.21563 16447247

[6] van der Heijde D Kivitz A Schiff MH Sieper J Dijkmans BAC Braun J. Efficacy and safety of adalimumab in patients with ankylosing spondylitis: results of a multicenter, randomized, double-blind, placebo-controlled trial. Arthritis Rheum. (2006) 54:2136–46. 10.1002/art.21913 16802350

[7] Curtis JR Mariette X Gaujoux-Viala C Blauvelt A Kvien TK Sandborn WJ. Long-term safety of certolizumab pegol in rheumatoid arthritis, axial spondyloarthritis, psoriatic arthritis, psoriasis and Crohn's disease: a pooled analysis of 11 317 patients across clinical trials. RMD Open. (2019) 5:e000942. 10.1136/rmdopen-2019-000942 31245056 PMC6560674

[8] Landewé R van der Heijde D Dougados M Baraliakos X Van den Bosch F Gaffney K. Induction of sustained clinical remission in early axial spondyloarthritis following certolizumab pegol treatment: 48-week outcomes from C-OPTIMISE. Rheumatol Ther. (2020) 7:581–99. 10.1007/s40744-020-00214-7 32529495 PMC7410911

[9] Braun J Baraliakos X Hermann KG Deodhar A van der Heijde D Inman D. The effect of two golimumab doses on radiographic progression in ankylosing spondylitis: results through 4 years of the GO-RAISE trial. Ann Rheum Dis. (2014) 73:1107–13. 10.1136/annrheumdis-2012-203075 23644549 PMC4033110

[10] van der Heijde D Deodhar A Braun J Mack M Hsu B Gathany TA. The effect of golimumab therapy on disease activity and health-related quality of life in patients with ankylosing spondylitis: 2-year results of the GO-RAISE trial. J Rheumatol. (2014) 41:1095–103. 10.3899/jrheum.131003 24737912

[11] Baraliakos X Kivitz AJ Deodhar AA Braun J Wei JC Delicha EM. Long-term effects of interleukin-17A inhibition with secukinumab in active ankylosing spondylitis: 3-year efficacy and safety results from an extension of the Phase 3 MEASURE 1 trial. Clin Exp Rheumatol. (2018) 36:50–5. 28516874

[12] Marzo-Ortega H Sieper J Kivitz A Blanco R Cohen M Delicha EM. Secukinumab provides sustained improvements in the signs and symptoms of active ankylosing spondylitis with high retention rate: 3-year results from the phase III trial, MEASURE 2. RMD Open. (2017) 3:e000592. 10.1136/rmdopen-2017-000592 29435364 PMC5761290

[13] van der Heijde D Song IH Pangan AL Deodhar A van den Bosch F Maksymowych WP. Efficacy and safety of upadacitinib in patients with active ankylosing spondylitis (SELECT-AXIS 1): a multicentre, randomised, double-blind, placebo-controlled, phase 2/3 trial. Lancet. (2019) 394:2108–17. 10.1016/S0140-6736(19)32534-6 31732180

[14] Wailoo A Bansback N Chilcott J. Infliximab, etanercept and adalimumab for the treatment of ankylosing spondylitis: cost-effectiveness evidence and NICE guidance. Rheumatology (Oxford). (2008) 47:119–20. 10.1093/rheumatology/kem294 18208819

[15] McLeod C Bagust A Boland A Dagenais P Dickson R Dundar Y. Adalimumab, etanercept and infliximab for the treatment of ankylosing spondylitis: a systematic review and economic evaluation. Health Technol Assess. (2007) 11:1–158, iii–iv. 10.3310/hta11280 17651658

[16] Lubrano E Perrotta FM Manara M D'Angelo S Ramonda R Punzi L. Improvement of function and its determinants in a group of axial spondyloarthritis patients treated with TNF inhibitors: a real-life study. Rheumatol Ther. (2020) 7:301–10. 10.1007/s40744-020-00197-5 32062827 PMC7211226

[17] Ungprasert P Erwin PJ Koster MJ. Indirect comparisons of the efficacy of biological agents in patients with active ankylosing spondylitis: a systematic review and meta-analysis. Clin Rheumatol. (2017) 36:1569–77. 10.1007/s10067-017-3693-7 28551823

[18] Lindström U Glintborg B Di Giuseppe D Nordström D Provan SA Gudbjornsson B Treatment retention of infliximab and etanercept originators vs. their corresponding biosimilars: Nordic collaborative observational study of 2334 biologics naïve patients with spondyloarthritis. RMD Open. (2019) 5:e001079 10.1136/rmdopen-2019-001079 31749988 PMC6827791

[19] Glintborg B Loft AG Omerovic E Hendricks O Linauskas A Espesen J. To switch or not to switch: results of a nationwide guideline of mandatory switching from originator to biosimilar etanercept. one-year treatment outcomes in 2061 patients with inflammatory arthritis from the DANBIO registry. Ann Rheum Dis. (2019) 78:192–200. 10.1136/annrheumdis-2018-213474 30396903

[20] Moots RJ Curiale C Petersel D Rolland C Jones H Mysler E. Efficacy and safety outcomes for originator TNF inhibitors and biosimilars in rheumatoid arthritis and psoriasis trials: a systematic literature review. BioDrugs. (2018) 32:193–9. 10.1007/s40259-018-0283-4 29790131

[21] Baraliakos X Østergaard M Gensler LS Poddubnyy D Lee EY Kiltz U. Comparison of the effects of secukinumab and adalimumab biosimilar on radiographic progression in patients with ankylosing spondylitis: design of a randomized, phase IIIb study (SURPASS). Clin Drug Investig. (2020) 40:269–78. 10.1007/s40261-020-00886-7 31983056

[22] Cantini F Niccoli L Benucci M Chindamo D Nannini C Olivieri I. Switching from infliximab to once-weekly administration of 50 mg etanercept in resistant or intolerant patients with ankylosing spondylitis: results of a fifty-four-week study. Arthritis Rheum. (2006) 55:812–6. 10.1002/art.22236 17013842

[23] van der Linden S Valkenburg HA Cats A. Evaluation of diagnostic criteria for ankylosing spondylitis. a proposal for modification of the New York criteria. Arthritis Rheum. (1984) 27:361–8. 10.1002/art.1780270401 6231933

[24] van der Heijde D Lie E Kvien TK Sieper J Van den Bosch F Listing J. ASDAS, a highly discriminatory ASAS-endorsed disease activity score in patients with ankylosing spondylitis. Ann Rheum Dis. (2009) 68:1811–8. 10.1136/ard.2008.100826 19060001

[25] Anderson JJ Baron G van der Heijde D Felson DT Dougados M. Ankylosing spondylitis assessment group preliminary definition of short-term improvement in ankylosing spondylitis. Arthritis Rheum. (2001) 44:1876–86. 10.1002/1529-0131(200108)44:8<;1876::AID-ART326>;3.0.CO;2-F 11508441

[26] Brandt J Listing J Sieper J Rudwaleit M van der Heijde D Braun J. Development and preselection of criteria for short term improvement after anti-TNF alpha treatment in ankylosing spondylitis. Ann Rheum Dis. (2004) 63:1438–44. 10.1136/ard.2003.016717 15044211 PMC1754796

[27] Wei JC Treat-to-target in spondyloarthritis: implications for clinical trial designs. Drugs. (2014) 74:1091–6. 10.1007/s40265-014-0246-0 24969316

[28] Ward MM Deodhar A Gensler LS Dubreuil M Yu D Khan MA Update of the American College of Rheumatology/Spondylitis Association of America/spondyloarthritis research and treatment network recommendations for the treatment ofankylosing spondylitis and nonradiographic axial spondyloarthritis. Arthritis Rheumatol. (2019) 71:1599–613. 10.1002/art.41042 31436036 PMC6764882

[29] Heinonen AV Aaltonen KJ Joensuu JT Lähteenmäki JP Pertovaara MI Romu MK. Effectiveness and drug survival of TNF inhibitors in the treatment of ankylosing spondylitis: a prospective cohort study. J Rheumatol. (2015) 42:2339–46. 10.3899/jrheum.150389 26472421

[30] Lie E van der Heijde D Uhlig T Mikkelsen K Rødevand E Koldingsnes W. Effectiveness of switching between TNF inhibitors in ankylosing spondylitis: data from the NOR-DMARD register. Ann Rheum Dis. (2011) 70:157–63. 10.1136/ard.2010.131797 21062852

[31] Glintborg B Østergaard M Krogh NS Tarp U Manilo N Gitte A. Clinical response, drug survival and predictors thereof in 432 ankylosing spondylitis patients after switching tumour necrosis factor alpha inhibitor therapy: results from the Danish nationwide DANBIO registry. Ann Rheum Dis. (2013) 72:1149–55. 10.1136/annrheumdis-2012-201933 22941767

[32] Deodhar A Yu D. Switching tumor necrosis factor inhibitors in the treatment of axial spondyloarthritis. Semin Arthritis Rheum. (2017) 47:343–50. 10.1016/j.semarthrit.2017.04.005 28551170

[33] Smolen JS Braun J Dougados M Emery P Fitzgerald O Helliwell P. Treating spondyloarthritis, including ankylosing spondylitis and psoriatic arthritis, to target: recommendations of an international task force. Ann Rheum Dis. (2014) 73:6–16. 10.1136/annrheumdis-2013-203419 23749611 PMC3888616

[34] Bolge SC Eldridge HM Lofland JH Ravin C Hart PJ Ingham MP. Patient experience with intravenous biologic therapies for ankylosing spondylitis, Crohn's disease, psoriatic arthritis, psoriasis, rheumatoid arthritis, and ulcerative colitis. Patient Prefer Adherence. (2017) 11:661–9. 10.2147/PPA.S121032 28405158 PMC5378465

[35] Ip K Hartley L Solanki K White D. Retention on anti-tumour necrosis factor therapy: the Waikato experience. N Z Med J. (2015) 128:34–40. 26117510

[36] Lindström U Olofsson T Wedren S Qirjazo Askling J. Impact of extra-articular spondyloarthritis manifestations and comorbidities on drug retention of a first TNF-inhibitor in ankylosing spondylitis: a population-based nationwide study. RMD Open. (2018) 4:e000762. 10.1136/rmdopen-2018-000762 30402269 PMC6203098

[37] Rudwaleit M Schwarzlose S Hilgert ES Listing J Braun J Sieper J. MRI in predicting a major clinical response to anti-tumour necrosis factor treatment in ankylosing spondylitis. Ann Rheum Dis. (2008) 67:1276–81. 10.1136/ard.2007.073098 18006539

[38] Yang R Liu H Fan M. A quick decrease of bone marrow edema in sacroiliac joint could be served as a novel marker for dose tapering of etanercept in ankylosing spondylitis patients. Medicine (Baltimore). (2019) 98:e14620. 10.1097/MD.0000000000014620 30882628 PMC6426528

[39] Soliman E El-Tantawi G Matrawy K Aldawoudy A Naguib A. Local infliximab injection of sacroiliac joints in non-radiographic axial spondyloarthritis: impact on clinical and magnetic resonance imaging parameters of disease activity. Mod Rheumatol. (2015) 25:421–6. 10.3109/14397595.2014.972495 25401227

[40] van der Heijde D Dougados M Weisman MH Maksymowych W Braun J. ASsessment in ankylosing spondylitis International working group/spondylitis association of America recommendations for conducting clinical trials in ankylosing spondylitis. Arthritis Rheum. (2005) 52:386–94. 10.1002/art.20790 15693009

[41] Mok CC van der Kleij D Wolbink GJ. Drug levels, anti-drug antibodies, and clinical efficacy of the anti-TNFα biologics in rheumatic diseases. Clin Rheumatol. (2013) 32:1429–35. 10.1007/s10067-013-2336-x 23887439

[42] Senabre Gallego JM Rosas J Marco-Mingot M García-Gómez JA Santos-Soler G Salas-Heredia E. Clinical relevance of monitoring serum adalimumab levels in axial spondyloarthritis. Rheumatol Int. (2019) 39:841–9. 10.1007/s00296-019-04288-7 30899987

[43] Wang W Leu J Watson R Xu Z Zhou H. Investigation of the mechanism of therapeutic protein-drug interaction between methotrexate and golimumab, an Anti-TNFα monoclonal antibody. AAPS J. (2018) 20:63. 10.1208/s12248-018-0219-4 29667047

[44] Lorenzin M Ortolan A Frallonardo P Oliviero F Punzi L Ramonda R. Predictors of response and drug survival in ankylosing spondylitis patients treated with infliximab. BMC Musculoskelet Disord. (2015) 16:166. 10.1186/s12891-015-0620-4 26205000 PMC4513706

[45] Lubrano E Perrotta FM Manara M D'Angelo S Addimanda O Ramonda R. The sex influence on response to tumor necrosis factor-α inhibitors and remission in axial spondyloarthritis. J Rheumatol. (2018) 45:195–201. 10.3899/jrheum.170666 29419448

[46] Scirè CA Caporali R Sarzi-Puttini P Frediani B Franco MD Tincani A. Drug survival of the first course of anti-TNF agents in patients with rheumatoid arthritis and seronegative spondyloarthritis: analysis from the monitorNet database. Clin Exp Rheumatol. (2013) 31:857–63. 23981363

