# Supplementary material for: Head-to-Head Comparison of Etanercept vs. Adalimumab in the Treatment of Ankylosing Spondylitis: An Open-Label Randomized Controlled Crossover Clinical Trial
Source: Front Med (Lausanne). 2020 Oct 30;7:566160. doi: 10.3389/fmed.2020.566160 (PMC7662505; doi:10.3389/fmed.2020.566160)
Supplement: Supplementary file 1 [file Data_Sheet_1.docx]

| Supplementary Table 1：Week4 vs Week0. | | | |
| --- | --- | --- | --- |
|  | Enbrel🡪Humira  (n = 9) | Humira🡪Enbrel  (n = 9) | *p* value |
| Weight (kg)^b^ | 0.50 (1.00)  60 | 0.00 (1.50) | 0.663 |
| Systolic blood pressure (mmHg)^a^ | 15.78 ± 18.71 | 2.33 ± 14.82 | 0.111 |
| Diastolic blood pressure (mmHg)^a^ | 8.44 ± 10.33 | 2.67 ± 8.41 | 0.212 |
| MBP ^a^ | 10.90 ± 12.66 | 2.56 ± 9.19 | 0.129 |
| WBC(10^3/uL)^a^ | -0.80 ± 1.49 | -1.05 ± 1.65 | 0.741 |
| RBC(10^4/uL)^a^ | 2.89 ± 25.82 | 7.00 ± 21.68 | 0.719 |
| Hb (gm/dl)^a^ | 0.20 ± 0.61 | 0.33 ± 0.83 | 0.703 |
| Ht(%)^a^ | 0.78 ± 2.36 | 0.84 ± 2.16 | 0.951 |
| MCV(fl)^b^ | 0.90 (1.10) | 0.20 (0.90) | 0.232 |
| MCH(Pg)^b^ | 0.20 (0.70) | 0.10 (0.60) | 0.862 |
| MCHC(g/dl)^b^ | -0.10 (0.70) | 0.00 (0.70) | 0.412 |
| Platelet(10^3/uL)^a^ | -45.56 ± 37.28 | -1.89 ± 96.19 | 0.232 |
| GPT (IU/l)^b^ | 3.00 (7.00) | 2.00 (4.00) | 0.571 |
| Creatinine (mg/dl)^a^ | 0.01 ± 0.11 | 0.02 ± 0.19 | 0.878 |
| HS-CRP(mg/dL^3^)^b^ | -0.25 (0.99) | -0.16 (1.61) | 0.850 |
| ESR (mm/hr)^b^ | -4 (23) | 0 (61) | 0.463 |
| IgA ^b^ | -20.00 (59.00) | -8.00 (15.00) | 0.794 |
| PGA ^a, c^ | -2.17 ± 2.22 | -1.11 ± 1.76 | 0.281 |
| PGA^a, d^ | -1.50 ± 2.67 | -1.89 ± 3.55 | 0.796 |
| BASDAI ^a^ | -1.47 ± 2.59 | -1.56 ± 1.44 | 0.929 |
| BASFI ^b^ | -1.10 (3.00) | -2.39 (4.20) | 0.489 |
| BAS-G ^a^ | -1.59 ± 3.08 | -2.64 ± 2.06 | 0.408 |
| ASDAScrp | -0.74 ± 1.32 | -1.02 ± 1.22 | 0.644 |
| ASDASesr | -0.54 ± 1.02 | -0.80 ± 0.70 | 0.545 |
| ^a^ mean ± SD, ^b^ median (IQR), ^c^PGA：Patient Global Assessment, ^d^ PGA：Physician Global Assessment | | | |

| Supplementary Table 2：Week8 vs Week0 | | | |
| --- | --- | --- | --- |
|  | Enbrel🡪Humira  (n = 9) | Humira🡪Enbrel  (n = 9) | *p* value |
| Weight (kg)^b^ | 0.50 (1.50) | 0.00 (1.80) | 0.825 |
| Systolic blood pressure (mmHg)^a^ | 3.44 ± 14.85 | 7.89 ± 11.91 | 0.494 |
| Diastolic blood pressure (mmHg)^a^ | 4.22 ± 10.46 | 7.33 ± 11.69 | 0.560 |
| MBP ^a^ | 3.97 ± 10.18 | 7.52 ± 11.20  0.492 | 0.492 |
| WBC(10^3/uL)^a^ | -1.01 ± 0.90 | -0.51 ± 1.01 | 0.281 |
| RBC(10^4/uL)^a^ | 0.22 ± 34.18 | 7.89 ± 26.74 | 0.603 |
| Hb (gm/dl)^a^ | 0.37 ± 1.00 | 0.51 ± 1.12 | 0.776 |
| Ht(%)^a^ | 0.82 ± 3.05 | 1.01 ± 2.42 | 0.886 |
| MCV(fl)^b^ | 1.60 (1.00) | 0.00 (3.00) | 0.130 |
| MCH(Pg)^b^ | 0.80 (0.40) | 0.10 (0.70) | 0.344 |
| MCHC(g/dl)^b^ | 0.20 (0.60) | 0.10 (0.90) | 1.000 |
| Platelet(10^3/uL)^a^ | -27.00 ± 53.64 | 13.78 ± 95.16 | 0.279 |
| GPT (IU/l)^b^ | 2.00 (7.00) | 6.00 (14.00) | 0.896 |
| Creatinine (mg/dl)^a^ | 0.01± 0.09 | 0.04 ± 0.14 | 0.565 |
| HS-CRP(mg/dL^3^)^b^ | -0.08 (1.00) | -0.21 (0.56) | 0.794 |
| ESR (mm/hr)^b^ | -10 (32) | -5 (14) | 1.000 |
| IgA ^b^ | -25.00 (100.00) | -4.00 (43.00) | 0.545 |
| PGA ^a, c^ | -2.89 ± 2.79 | -1.73 ± 1.40 | 0.283 |
| PGA^a, d^ | -1.44 ± 4.10 | -1.44 ± 2.01 | 1.000 |
| BASDAI ^a^ | -1.59 ± 3.07 | -2.20 ± 2.44 | 0.648 |
| BASFI ^b^ | -1.10 (3.60) | -2.20 (3.75) | 0.389 |
| BAS-G ^a^ | -2.06 ± 3.45 | -2.78 ± 1.97 | 0.596 |
| ASDAScrp | -0.90 ± 1.39 | -1.24 ± 1.40 | 0.612 |
| ASDASesr | -0.67 ± 1.03 | -0.96 ± 1.01 | 0.558 |
| ^a^ mean ± SD, ^b^ median (IQR), ^c^PGA：Patient Global Assessment, ^d^ PGA：Physician Global Assessment | | | |

| Supplementary Table3：Week12 vs Week0 | | | |
| --- | --- | --- | --- |
|  | Enbrel🡪Humira  (n = 9) | Humira🡪Enbrel  (n = 9) | *p* value |
| Weight (kg)^b^ | 1.00 (1.50) | 1.00 (1.50) | 0.826 |
| Systolic blood pressure (mmHg)^a^ | 9.00 ± 20.91 | 0.00 ± 8.57 | 0.258 |
| Diastolic blood pressure (mmHg)^a^ | 10.22 ± 13.66 | 0.67 ± 14.25 | 0.166 |
| MBP ^a^ | 9.82 ± 12.97 | 0.44 ± 11.27 | 0.121 |
| WBC(10^3/uL)^a^ | -0.77 ± 1.42 | -0.74 ± 1.31 | 0.969 |
| RBC(10^4/uL)^a^ | 15.44 ± 42.47 | 0.67 ± 27.97 | 0.396 |
| Hb (gm/dl)^a^ | 0.77 ± 1.30 | 0.51 ± 1.26 | 0.678 |
| Ht(%)^a^ | 2.16 ± 3.94 | 0.91 ± 2.94 | 0.458 |
| MCV(fl)^b^ | 1.40 (1.90) | 0.40 (1.40) | 0.163 |
| MCH(Pg)^b^ | 0.60 (0.90) | 0.10 (0.70) | 0.516 |
| MCHC(g/dl)^b^ | 0.30 (0.30) | 0.10 (1.20) | 0.965 |
| Platelet(10^3/uL)^a^ | **-**13.11± 68.85 | 0.89 ± 97.13 | 0.729 |
| GPT (IU/l)^b^ | 7.00 (5.00) | 5.00 (12.00) | 0.284 |
| Creatinine (mg/dl)^a^ | 0.01 ± 0.15 | 0.03 ± 0.12 | 0.730 |
| HS-CRP(mg/dL^3^)^b^ | -0.42 (1.04) | -0.18 (0.50) | 0.896 |
| ESR (mm/hr)^b^ | -7 (28) | -4 (17) | 0.694 |
| IgA ^b^ | -8.00 (63.00) | -25.00 (57.00) | 0.761 |
| PGA ^a, c^ | -3.22 ± 2.58 | -1.68 ± 1.58 | 0.144 |
| PGA^a, d^ | -1.89 ± 3.41 | -1.56 ± 2.24 | 0.810 |
| BASDAI ^a^ | -2.18 ± 2.87 | -1.69 ± 2.46 | 0.704 |
| BASFI ^b^ | -1.10 (3.65) | -1.00 (3.21) | 0.761 |
| BAS-G ^a^ | -2.70 ± 3.10 | -2.47 ± 2.25 | 0.861 |
| ASDAScrp | -1.08 ± 1.31 | -1.12 ± 1.45 | 0.952 |
| ASDASesr | -0.80 ± 0.99 | -0.83 ± 0.99 | 0.948 |
| ^a^ mean ± SD, ^b^ median (IQR), ^c^PGA：Patient Global Assessment, ^d^ PGA：Physician Global Assessment | | | |

| Supplementary Table4：Week16 vs Week0 | | | |
| --- | --- | --- | --- |
|  | Enbrel🡪Humira  (n = 9) | Humira🡪Enbrel  (n = 9) | *p* value |
| Weight (kg)^b^ | 0.00 (3.90) | 0.50 (2.23) | 0.776 |
| Systolic blood pressure (mmHg)^a^ | 6.22 ± 16.37 | 2.56 ± 13.12 | 0.607 |
| Diastolic blood pressure (mmHg)^a^ | 4.33 ± 8.31 | 9.11 ± 9.33 | 0.773 |
| MBP ^a^ | 4.97 ± 9.12 | 2.93 ± 9.59 | 0.649 |
| WBC(10^3/uL)^a^ | -0.58 ± 1.86 | 0.12 ± 1.59 | 0.409 |
| RBC(10^4/uL)^a^ | 12.11 ± 34.72 | 5.44 ± 21.81 | 0.632 |
| Hb (gm/dl)^a^ | -0.40 ± 2.84 | 0.82 ± 1.52 | 0.272 |
| Ht(%)^a^ | 2.16 ± 3.94 | 0.91 ± 2.94 | 0458 |
| MCV(fl)^b^ | 1.40 (3.40) | 0.10 (1.50) | 0.176 |
| MCH(Pg)^b^ | 0.60 (1.10) | 0.30 (0.70) | 0.828 |
| MCHC(g/dl)^b^ | 0.20 (1.10) | 0.20 (0.90) | 0.463 |
| Platelet(10^3/uL)^a^ | -24.00 ± 59.62 | 11.33 ± 106.83 | 0.399 |
| GPT (IU/l)^b^ | 8.00 (10.00) | 6.00 (17.00) | 1.000 |
| Creatinine (mg/dl)^a^ | 0.00 ± 0.12 | 0.08 ± 0.11 | 0.174 |
| HS-CRP(mg/dL^3^)^b^ | -0.31 (1.10) | -0.17 (0.51) | 0.965 |
| ESR (mm/hr)^b^ | -7 (29) | -2 (13) | 0.794 |
| IgA ^b^ | -17.00 (61.00) | 0.00 (47.00) | 0.390 |
| PGA ^a, c^ | -3.33 ± 2.60 | -1.79 ± 1.39 | 0.135 |
| PGA^a, d^ | -2.94 ± 3.07 | -1.72 ± 2.31 | 0.354 |
| BASDAI ^a^ | -2.25 ± 2.61 | -2..30 ± 2.40 | 0.968 |
| BASFI ^b^ | -1.10 (2.91) | -1.10 (2.80) | 0.965 |
| BAS-G ^a^ | -2.62 ± 3.12 | -2.58 ± 2.13 | 0.976 |
| ASDAScrp | -1.02 ± 1.22 | -1.26 ± 1.44 | 0.707 |
| ASDASesr | -0.78 ± 0.90 | -0.96 ± 1.02 | 0.697 |
| ^a^ mean ± SD, ^b^ median (IQR), ^c^PGA：Patient Global Assessment, ^d^ PGA：Physician Global Assessment | | | |
