# Supplementary material for: Head-to-Head Comparison of Etanercept vs. Adalimumab in the Treatment of Ankylosing Spondylitis: An Open-Label Randomized Controlled Crossover Clinical Trial
Source: Front Med (Lausanne). 2020 Oct 30;7:566160. doi: 10.3389/fmed.2020.566160 (PMC7662505; doi:10.3389/fmed.2020.566160)
Supplement: Supplementary file 2 [file Data_Sheet_2.doc]

**Etanercept versus Adalimumab in the Treatment of Patients with Ankylosing Spondylitis.**

**A Switch Study.**

James Cheng-Chung Wei, M.D., Ph.D.

Division of Allergy, Immunology and Rheumatology; Clinical Trial Center

Chung Shan Medical University Hospital

Taichung, Taiwan.

No. 110, Sec. 1, Jianguo N. Rd., South District, Taichung City 40201, Taiwan
[wei3228@gmail.com](mailto:wei3228@gmail.com)

(TEL)04 24739595 #33410 (FAX) 04 24739220

(Mobile) +886 4  931227420

# SYNOPSIS

**Study Title**

Etanercept versus Adalimumab in the Treatment of Patients with Ankylosing Spondylitis.

A Switch Study.

**Clinical Phase** 4

**Study Backgrounds**

Ankylosing spondylitis (AS) is a chronic inflammatory arthritis causing back pain, peripheral arthritis and enthesitis. Anti-tumor necrosis factor (TNF) biological agents, including etanercept[1, 2], infliximab[3, 4] and adalimumab[5, 6] had been proved to have dramatic anti-inflammatory and immunomodulatory effect in AS. Infliximab, etanercept and adalimumab are also cost-effective in the treatment of ankylosing spondylitis.[7, 8]

Aanti-TNF therapies have been proved very dramatic efficacy in the treatment of AS, with a 50% reduction of disease activity in about 60% of patients. However, 20% and 30%, are nonresponders or achieve only a partial response. Moreover, in an additional 10-20% of patients, the treatment should be interrupted due to loss of efficacy or side effects over the followup period. In the case of treatment failure with one agent, switching to the other agent may also be useful in patients with AS due to the different chemical structures and mechanisms of action of drugs.

In rheumatoid arthritis (RA), about 20% of patients might not respond to one anti-TNF therapy. Switch studies from one anti-TNF agent to another anti-TNF agent have been done. A systematic review[9] included 33 switch studies in RA showed that most frequently switches are those between etanercept and infliximab. The results are promising but not excellent. Any switch, especially those between monoclonal antibodies, have an effect size that is usually lower than that of a first biologic. When the switch is due to an adverse event with the first TNF-antagonist, however, the response rate of the second one is high. [9]

In a cohort of 282 patients with rheumatoid arthritis treated with infliximab or etanercept, response after switching was recorded as a success for 18 out of the 24 patients who were treated with infliximab first, and for 12 out of the 14 patients who were treated with etanercept first. Among the 29 patients who discontinued the first anti TNF-alpha treatment due to lack of efficiency, only 6 did not respond to the second anti TNF-alpha treatment.[10] A randomised, open-label, clinical trial of 28 RA patients with an inadequate response to etanercept were randomised 1:1 to discontinue etanercept and receive infliximab 3 mg/kg, or to continue etanercept 25 mg twice weekly. At week 16, 62% of infliximab-treated patients achieved American College of Rheumatology 20% criteria for improvement in RA (ACR20) responses compared with 29% of etanercept-treated patients. A 30.8% decrease from baseline in Disease Activity Score 28 was observed in patients receiving infliximab, compared with a 16.0% decrease in patients receiving etanercept. [11]

For spondyloarthropathy (SpA) and psoriatic arthritis (PsA), one report of switching from infliximab to etanercept because of inadequate response or adverse events demonstrated that 9 of 13 patients responded to etanercept and none experienced intolerance to this agent. They suggest that switching between anti-TNF-alpha drugs may be useful for patients with SpA who are unresponsive or intolerant to a first anti-TNF-alpha agent. [12]

For ankylosing spondylitis (AS), a switching study from infliximab to etanercept showed good response for patients who are resistant or intolerant to infliximab. Switching to etanercept after infliximab treatment escape restores the clinical response in the majority of patients. Moreover, etanercept, administered at a dosage of 50 mg once weekly, is effective and well tolerated in patients with AS.[13]. However, no switch study from etanercept to adalimumab was done so far. Thus, we conducted this open label randomized controlled study in etanercept-treated AS patients to analyze the effect of switch to adalimumab.

**Study Objective**

To determine the safety and efficacy of switch effects to adalimumab in etanercept-treated AS patients.

**Study Design**

This single-centre, open-labeled randomized controlled study will evaluate the safety and efficacy of adalimumab in etanercept-treated AS patients. Thirty patients will be enrolled and randomized equally into two arms. The treatment arm will receive adalimumab 40 mg subcutaneously biweekly for 8 weeks. The control arm will continue etanercept 25 mg subcutaneously twice a week for 8 weeks. At week 8, the control arm will be switched to adalimumab 40 mg subcutaneously biweekly for another 8 weeks. Evaluations will be performed at baseline, weeks 4, 8, 12 and 16**.**

**Patients**

**Inclusion criteria**

1. Diagnosis of AS, as defined by 1984 Modified New York Criteria for Ankylosing Spondylitis
2. Stable background therapy as non-steroid anti-inflammatory for 2 weeks, glucocorticoid for 4 weeks, disease-modifying anti-rheumatic drugs, eg. sulphasalazine, and methotrexate for 8 weeks, anti-TNF biologics for 4 weeks.
3. Written informed consent.

**Exclusion criteria**

1. Serum creatinine ≥ 3.0 mg/dl;
2. Alanine aminotransferase (ALT [SGPT]) ≥ 5 times the laboratory’s upper limit of normal.
3. Pregnant or breast-feeding women.

**Duration of Study:** 16 weeks.

**Number of Subjects** 30

**Number of Study Centers** 1

**Dosage and Administration**

For standard dose（etanercept 50 mg weekly） treated patients, the treatment arm will receive adalimumab 40 mg subcutaneously biweekly for 8 weeks. The control arm will continue etanercept 25 mg subcutaneously twice a week for 8 weeks. At week 8, the control arm will be switched to adalimumab 40 mg subcutaneously biweekly for another 8 weeks.

For half dose （etanercept 25 mg weekly） treated patients, the treatment arm will receive adalimumab 40 mg subcutaneously monthly for 8 weeks. The control arm will continue etanercept 25 mg subcutaneously weekly for 8 weeks. At week 8, the control arm will be switched to adalimumab 40 mg subcutaneously monthly for another 8 weeks.

**Equivalent dose**

etanercept 50 mg weekly = adalimumab 40 mg biweekly

etanercept 25 mg weekly = adalimumab 40 mg monthly

**Concomitant Treatment** All concomitant treatments (including NSAIDs and corticosteroids) will be recorded on the patient’s case report form including the name of the drug (generic names only except for combination products), total daily dose, and duration of treatment. Additionally, for corticosteroids and NSAIDs, route of administration will be recorded.

**Prohibited:**

1. Any new treatment with a DMARD or a cytotoxic (immunosuppressive) drug except for hydroxychloroquine, sulphasalazine, and methotrexate
2. Surgical joint interventions (open or arthroscopic) .

**Efficacy Evaluation**

**Primary Efficacy Endpoint:**

- The primary endpoint of this study is Bath AS disease activity index (BASDAI) at week 8.

**Secondary Efficacy Endpoints will include:**

- ASAS20 response rate
- Mean change in each of the following domains:
  - VAS patient global assessment
  - VAS pain score
  - Bath AS functional index, BASFI
  - Bath AS global index, BAS-G
  - BASDAI –two morning stiffness-related scores
- Patient global assessment, physician global assessment, nocturnal and total back pain, the BASFI and its independent components, and the BASDAI and its independent components.
- Spinal mobility measured with modified Schober’s test, chest expansion, and occiput-to-wall measurement.
- Patients’ preference scale.
- Quality of life as ASQoL questionnaire.
- Laboratory assessment of inflammation with HS-CRP, Immunoglobulin A and ESR
- Serum creatinine、ALT (SGPT)、CBC

**Safety Evaluation** All patients who receive at least 1 dose of test medicine will be evaluated for safety. The following variables will be assessed: physical examination/vital signs, haematology and chemistry profiles, urinalysis, assessment of extra-spinal and extra-articular involvement, premature withdrawal, adverse events, and serious adverse events during the study.

**Statistical Analysis** Descriptive statistics such as number of observations, mean, median, standard deviation, minimum, maximum and 95% confidence interval will be used to summarize the continuous variables. Frequency and proportion will be used to summarize the categorical variables. The primary analysis will be performed based on the intent-to-treat population. All statistical tests will be two-sided which was evaluated at 5% level significance.

Frequency of dropouts, premature termination of study medication, and withdrawal will be provided and summarized. The last-observation-carried-forward (LOCF) approach will be used to evaluate missing data.

Summary tables for demographic data and baseline characteristics will be provided.

The primary efficacy endpoint is the number of responders as determined by the ASAS Response Criteria for improvement at 20% level at week 8. All patients who withdraw before 8 weeks will be considered non-responders for this endpoint.

Changes (and percentage changes) from baseline at each visit in the individual components of the ASAS Working Group criteria (VAS patient global assessment, VAS physician global assessment, VAS total and nocturnal pain, BASFI, BASDAI and BAS-G), spinal mobility measures, complete joint assessment, evaluation of hip involvement, and laboratory assessments of inflammation will be analyzed by using paired-t test.

The number and percentage of patients who experience adverse events will be summarized. The number and percentage of patients who experience potentially clinically significant changes in laboratory results, and those who discontinue, will be tabulated. Transition tables from baseline for physical examination and laboratory results will be summarized.

**Rationale for Number of Subjects** Assuming that the BASDAI scores of treatment group (μ1) reduced to 2 from 4, and the BASDAI scores of control group (μ0) reduce to 3.5 from 4. A 2-side test was used, with type Ⅰ error (α) = 0.05, statistical power (1-β) = 0.8 and standard deviation (SD) = 2. The expected dropout rate is 10% in this research. Finally, the study required only 14 subjects in each group to achieve 95% power. The targeted sample size for the study was 30 patients. Therefore, the study had > 95% power to detect a treatment effect with this sample size.

**Ethical Considerations** This study will be conducted in accordance with applicable laws and regulations including, but not limited to, the International Conference on Harmonization Guideline for Good Clinical Practice (GCP) and the ethical principles that have their origins in the Declaration of Helsinki. The institutional review board (IRB)/independent ethics committee (IEC) must review and approve the protocol and informed consent form (ICF) before any subjects are enrolled. The subject must be consented using the approved ICF before any procedures specified in the protocol are performed.

# STUDY FLOWCHART

Abbreviations: BASFI = Bath Ankylosing Spondylitis Functional Index; BASDAI = Bath Ankylosing Spondylitis Disease Activity Index. BAS-G = Bath Ankylosing Spondylitis Global Index.

| **Study Procedures** | **Week**  **-4** | **Week**  **0** | **Week 4** | **Week 8** | **Week 12** | **Week 16** |
| --- | --- | --- | --- | --- | --- | --- |
| Visit ID (for sponsor use only) | 1 | 2 | 3 | 4 | 5 | 5 |
| Signed informed consent | X |  |  |  |  |  |
| Medical history | X |  |  |  |  |  |
| Medical history update |  | X |  |  |  |  |
| Inclusion/exclusion criteria | X | X |  |  |  |  |
| Record prior medications | X |  |  |  |  |  |
| Record concomitant medications |  | X | X | X | X | X |
| Physical examination |  |  |  |  |  |  |
| Vital signs |  | X | X | X | X | X |
| Complete joint assessment |  | X | X | X | X | X |
| Patient global assessment |  | X | X | X | X | X |
| Physician global assessment |  | X | X | X | X | X |
| VAS pain score |  | X | X | X | X | X |
| Nocturnal and total back pain |  | X | X | X | X | X |
| BASFI | X | X | X | X | X | X |
| BASDAI | X | X | X | X | X | X |
| BAS-G | X | X | X | X | X | X |
| Modified Schober’s test |  | X | X | X | X | X |
| Chest expansion measurement |  | X | X | X | X | X |
| Occiput-to-wall distance |  | X | X | X | X | X |
| Serum creatinine | X | X |  |  | X | X |
| ALT (SGPT) | X | X |  |  | X | X |
| Erythrocyte sedimentation rate |  | X |  |  | X | X |
| HS-C-Reactive protein |  | X |  |  | X | X |
| Adverse event evaluation |  | X | X | X | X | X |
| Dispense diary cards |  | X | X | X | X | X |
| Dispense test article |  | X | X | X | X | X |
| BP、BW |  | X | X | X | X | X |
| CBC |  | X | X | X | X | X |
| IgA |  | X | X | X | X | X |
| ASQOL |  | X | X | X | X | X |
| finger to floor test |  | X | X | X | X | X |
| L’t lateral bending |  | X | X | X | X | X |
| Shoulder pain or limitation of motion |  | X | X | X | X | X |
| CONCOMITANT MEDICATION |  | X | X | X | X | X |
| ASAS Criteria for response |  |  | X | X | X | X |
| Patients’ preference scale |  |  | X |  |  | X |

The control group: (n = 15)

Etanercept 50 mg sc. weekly

Ankylosing Spondylitis

The treatment group: (n = 15)

Adalimumab 40 mg sc. biweekly

N=30

**Visit 1 Visit 2 Visit 3 Visit 4 Visit 5**

**(0 week) (4 weeks) (8 weeks) (12 weeks) (16 weeks)**

**References**

1. Brandt, J., et al., *Six-month results of a double-blind, placebo-controlled trial of etanercept treatment in patients with active ankylosing spondylitis.* Arthritis Rheum, 2003. **48**(6): p. 1667-75.

2. Davis, J.C., Jr., et al., *Recombinant human tumor necrosis factor receptor (etanercept) for treating ankylosing spondylitis: a randomized, controlled trial.* Arthritis Rheum, 2003. **48**(11): p. 3230-6.

3. Brandt, J., et al., *Successful treatment of active ankylosing spondylitis with the anti-tumor necrosis factor alpha monoclonal antibody infliximab.* Arthritis Rheum, 2000. **43**(6): p. 1346-52.

4. Braun, J., et al., *Treatment of active ankylosing spondylitis with infliximab: a randomised controlled multicentre trial.* Lancet, 2002. **359**(9313): p. 1187-93.

5. Haibel, H., et al., *Adalimumab reduces spinal symptoms in active ankylosing spondylitis: clinical and magnetic resonance imaging results of a fifty-two-week open-label trial.* Arthritis Rheum, 2006. **54**(2): p. 678-81.

6. van der Heijde, D., et al., *Efficacy and safety of adalimumab in patients with ankylosing spondylitis: results of a multicenter, randomized, double-blind, placebo-controlled trial.* Arthritis Rheum, 2006. **54**(7): p. 2136-46.

7. Wailoo, A., N. Bansback, and J. Chilcott, *Infliximab, etanercept and adalimumab for the treatment of ankylosing spondylitis: cost-effectiveness evidence and NICE guidance.* Rheumatology (Oxford), 2008. **47**(2): p. 119-20.

8. McLeod, C., et al., *Adalimumab, etanercept and infliximab for the treatment of ankylosing spondylitis: a systematic review and economic evaluation.* Health Technol Assess, 2007. **11**(28): p. 1-158, iii-iv.

9. Carmona, L., A. Ortiz, and M.A. Abad, *How good is to switch between biologics? A systematic review of the literature.* Acta Reumatol Port, 2007. **32**(2): p. 113-28.

10. Cohen, G., et al., *The efficiency of switching from infliximab to etanercept and vice-versa in patients with rheumatoid arthritis.* Clin Exp Rheumatol, 2005. **23**(6): p. 795-800.

11. Furst, D.E., et al., *Open-label, pilot protocol of patients with rheumatoid arthritis who switch to infliximab after an incomplete response to etanercept: the opposite study.* Ann Rheum Dis, 2007. **66**(7): p. 893-9.

12. Delaunay, C., et al., *Infliximab to etanercept switch in patients with spondyloarthropathies and psoriatic arthritis: preliminary data.* J Rheumatol, 2005. **32**(11): p. 2183-5.

13. Cantini, F., et al., *Switching from infliximab to once-weekly administration of 50 mg etanercept in resistant or intolerant patients with ankylosing spondylitis: results of a fifty-four-week study.* Arthritis Rheum, 2006. **55**(5): p. 812-6.

**計畫執行之相關人員**

| 類別 | 姓名 | 現職 | 本計劃內擔任之具體工作性質 |
| --- | --- | --- | --- |
| 計畫主持人 | 魏正宗 | 中山醫學大學附設醫院過敏免疫風濕科主任、中藥臨床試驗中心主任 | 計畫規劃、計畫執行、收集病人、西醫評估、論文撰寫 |
| 護理長 | 江郡瑜 | 中山醫學大學中藥臨床試驗中心 | 督導監測研究計畫執行 |
| 專任研究助理 | 廖珮珊 | 中山醫學大學中藥臨床試驗中心 | 協助研究計畫執行 |
| 專任研究助理 | 藍培熏 | 中山醫學大學中藥臨床試驗中心 | 協助研究計畫執行 |
| 兼任研究助理 | 許立潔 | 中山醫學大學中藥臨床試驗中心 | 協助研究計畫執行 |
| 專任研究助理 | 張淑媛 | 中山醫學大學中藥臨床試驗中心 | 協助研究計畫執行 |
| 藥劑師 | 蔡雅婷 | 中山醫學大學中藥臨床試驗中心 | 協助研究計畫執行 |
| 臨床統計師 | 李名鏞 | 中山醫學大學中藥臨床試驗中心 | 資料統計分析 |

**研究執行機構**

中山醫學大學附設醫院 過敏免疫風濕科、中藥臨床試驗中心

| **Study Budget** | **NTD** |
| --- | --- |
|  |  |
| **Study nurse** | **180000** |
| **Statistics** | **20000** |
| **Laboratory** | **100000** |
| **IRB fee** | **60000** |
| **Administration fee** | **40000** |
|  |  |
| **Total** | **400000** |
